# The Mediating Role of Misinterpretations and Neutralizing Responses to Unwanted Intrusive Thoughts in Obsessive-Compulsive Spectrum Disorders

**DOI:** 10.3390/ejihpe15070135

**Published:** 2025-07-15

**Authors:** Belén Pascual-Vera, Guy Doron, Mujgan Inozu, Fernando García, Amparo Belloch

**Affiliations:** 1 Department of Personality Psychology, National University of Distance Education (UNED), C/Juan Rosal, 10, 28040 Madrid, Spain; 2 Baruch Ivcher School of Psychology, Reichman University (IDC), Herzliya 4610101, Israel; gdoron@idc.ac.il; 3 Department of Psychology, Hacettepe University, Ankara 06909, Turkey; mujganinozu@hacettepe.edu.tr; 4 Aiglé Foundation, Buenos Aires 1426, Argentina; fernandosebastiang17@gmail.com; 5 Personality, Evaluation and Psychological Treatment Department, University of Valencia, 46010 Valencia, Spain

**Keywords:** obsessive-compulsive disorder, body dysmorphic disorders, hypochondriasis, illness anxiety disorder, intrusive cognitions, cognitive behavioral therapy, mediation analysis

## Abstract

**Background.** Cognitive-behavioral theories suggest that obsessions in obsessive-compulsive disorder (OCD) develop from maladaptive misinterpretations and coping strategies of unwanted intrusive thoughts (UITs). Models of Body Dysmorphic Disorder (BDD) and Illness Anxiety Disorder (IAD) propose that these symptoms stem from similar misinterpretations of common UITs relating to perceived defects in appearance and illness. This study examines whether maladaptive misinterpretations and control strategies leading to the escalation of obsessional UITs to OCD symptoms also have a similar effect on the development of BDD and IAD. More specifically, we examined whether misinterpretations and neutralizing responses mediate the associations between the frequency of disorder-specific UITs and symptoms of these disorders. **Method.** A total of 625 non-clinical participants from four countries completed the Questionnaire of Unpleasant Intrusive Thoughts (QUIT) that assesses OCD, BDD and IAD-related UITs and their associated misinterpretations and neutralizing strategies, as well as self-report measures of OCD, BDD, and IAD symptoms. Parallel multiple mediation models were conducted. **Results**. The frequency of OCD, BDD and IAD-related UITs predicted symptoms of each disorder. Dysfunctional appraisals and neutralizing behaviors mediated the associations between disorder-specific UITs and symptoms in OCD and IAD. The IAD model accounted for a smaller proportion of variance than the OCD model. No mediating effects were found for BDD symptoms. **Conclusions**. Experiencing disturbing UITs is a transdiagnostic risk factor of OCD, BDD and IAD, and is associated with symptoms of these disorders. Maladaptive interpretation of UITs and neutralizing strategies should be specific targets in the assessment and treatment of OCD and IAD. The absence of mediation effects for BDD could be due to the limitations observed on the self-report used to assess BDD symptoms and/or the low relevance of the misinterpretations and control strategies assessed by the QUIT, which are more typically endorsed by individuals with OCD.

## 1. Introduction

Obsessive-Compulsive Disorder (OCD) is characterized by recurrent and persistent unwanted thoughts, images, and urges (i.e., obsessions) and behavioral or mental acts (i.e., compulsions) to keep obsessions under control. Both obsessions and compulsions interfere with daily activities and produce high levels of distress. Body Dysmorphic Disorder (BDD) is characterized by persistent preoccupations with one or more perceived defects or flaws in appearance that are either unnoticeable or only slightly noticeable to others (WHO, 2024). These appearance-related preoccupations cause significant distress and impairment and lead the individual to perform time-consuming and counterproductive behaviors (Eisen et al., 2004). Such behaviors are often repetitive and include appearance checking and reassurance seeking performed to control the distressing preoccupations (Veale & Neziroglu, 2010). Illness Anxiety Disorder (IAD) is defined by persistent preoccupation with having or acquiring a serious, progressive or life-threatening illness (APA, 2022). Such preoccupations are frequently accompanied by repetitive and/or excessive health-related behaviors, such as checking of the body for evidence of illness, searching for information about the feared illness, or repeatedly seeking reassurance (e.g., arranging multiple medical consultations). Although the name of the disorder in the ICD-11 (WHO, 2024) is health anxiety disorder or hypochondriasis, the main diagnostic and clinical features are almost identical to the ones in the DSM-5-TR for IAD.

The three disorders differ in important aspects that go beyond the differences in the content of their cognitions and behaviors. For instance, content and occurrence-related egodystonicity of intrusive thoughts in OCD seems to be higher than in appearance-related thoughts. Obsessional themes often contradict important aspects of self to a greater degree than do the appearance-related (Rowa & Purdon, 2003). Another important difference between OCD and BDD is the role that doubt plays in their pathogenesis. Individuals with BDD strongly believe that they look abnormal and “ugly” (Phillips et al., 2007). By contrast, individuals with OCD often doubt their perceptions and thoughts, particularly when the obsessions are associated with checking or washing compulsions (Clark & O’Connor, 2005). Insight about the disorder is another key difference between BDD and OCD. Although the insight of individuals with OCD fluctuates, subjects with BDD have generally poorer insight than those with OCD and, consequently, are more likely to be delusional. Suicidal ideation, lifetime major depression, and substance use disorder are also more likely in individuals with BDD than in those with OCD (Phillips et al., 2007). Notable differences are also found between OCD and IAD symptoms. IAD-related thoughts are specifically focused on the fear of having or developing a serious illness, whereas individuals with OCD experience a broader range of obsessive thoughts. Furthermore, individuals with IAD tend to experience a higher frequency of image-based UITs (e.g., Wells & Hackmann, 1993; Muse et al., 2010), greater fear of bodily sensations and lower insight into the irrational nature of their fears compared to those with OCD (e.g., Abramowitz & Braddock, 2006).

Despite the differences between the three disorders, all three show a similar functional relationship between obsessions and compulsive behaviors. These similarities, along with other commonalities, such as comorbidity, course, and age of onset (e.g., Frare et al., 2004; Phillips et al., 2007; Solem et al., 2015), support the current inclusion of BDD and IAD within the diagnostic section of obsessive compulsive and related disorders in the 11th edition of the International Classification of Diseases (WHO, 2024). Additionally, evidence suggests that these disorders respond similarly to pharmacological and psychological treatments, exhibit high comorbidity, and share similarities in their course (Abramowitz & Jacoby, 2015). Such similarities have been postulated as the basis for including BDD in the Obsessive-Compulsive and Related Disorders in the 5th revised version of the Diagnostic and Statistical Manual of Mental Disorders (APA, 2022).

The similarities among these three disorders can also be found in their cognitive-behavioral formulations. OCD related cognitive-behavioral theories trace the origin of obsessions to the experience of unwanted intrusive thoughts (UITs) involving various content domains (injury/harm, religion, sexuality, contamination, dirtiness, etc.). As occurs in OCD, recurrent thinking patterns that are often unacceptable or undesirable, difficult to diminish, and highly uncontrollable (i.e., Clark, 2005; Rachman, 1997) have also been described in BDD (Giraldo-O’Meara & Belloch, 2018; Osman et al., 2004) and in IAD (Muse et al., 2010; Wells & Hackmann, 1993). In the case of BDD, the contents of UITs are related to a perceived or slight defect in one’s appearance, while in IAD, they focus on having or acquiring a serious illness.

OCD-related UITs with contents analogous to clinical obsessions have been found in non-clinical populations (i.e., Clark, 2005; Rachman, 1997; Salkovskis, 1985) across different cultural and social contexts. Similar findings have been reported for appearance and illnesses related to UITs (Pascual-Vera et al., 2019, 2022). Data from these studies support the universality and transdiagnostic nature of UITs (e.g., Harvey et al., 2004). However, further investigation is needed to explore the role of various cognitive behavioral features in the escalation of common UITs into clinically significant UITs. Several aspects of UITs have been suggested, being their frequency and associated emotional distress the two most relevant, as they elicit misinterpretations and efforts to neutralize and/or control them (Berry & Laskey, 2012; Clark et al., 2000). In the case of OCD-related UITs, individuals tend to consider their UITs as more threatening and meaningful than those without OCD. This may be because they experience the content of their thoughts as unacceptable or immoral. They also fear that their thoughts may become true, and place greater importance on controlling their UITs. This may lead them to exert more effort in trying to “*do something*” about it, rather than simply ignoring these thoughts. According to cognitive-behavioral models of OCD (e.g., Clark, 2004; Rachman, 1997; Salkovskis, 1985), the interrelationships between maladaptive appraisals, emotional reactions, and maladaptive behaviors lead to the escalation of commonly occurring UITs to distressing obsessions. Specifically, these models suggest that an individual’s maladaptive appraisal of UITs (based on their personal values) leads them to engage in a variety of counterproductive neutralizing behaviors (i.e., thought control strategies, overt/covert behaviors, and reassurance, among others) to manage these thoughts. The transition from universal experiences to pathology, therefore, would depend on the maladaptive behavioral and emotional consequences (i.e., sadness, anxiety, guilt, shame) elicited by UITs. These assumptions have been largely supported by research with the general population and patients with OCD (i.e., Abramowitz et al., 2009; García-Soriano et al., 2011; OCCWG, 2001, 2003).

Similarly, cognitive-behavioral models of BDD (e.g., Fang & Wilhelm, 2015; Wilhelm et al., 2013) and IAD (e.g., Fergus, 2013; Marcus et al., 2007) suggest that clinically significant symptoms of these disorders evolve from UITs experienced by most people: UITs related to body defects in BDD, and UITs related to illness-death in IAD. Indeed, preoccupations with appearance-related defects and body image concerns are nearly universal, and they are probably a consequence of social normative pressures about one’s figure and appearance (Striegel-Moore & Bulik, 2007). These pressures are quite relevant during adolescence; in fact, BDD typically begins in early adolescence (Phillips et al., 2006). The universality of preoccupations about physical appearance defects suggests a dimensionality of these thoughts on a continuum ranging from normal concerns regarding one’s appearance to BDD-related preoccupations (Wilhelm & Neziroglu, 2002). In individuals with BDD, Giraldo-O’Meara and Belloch (2018) demonstrated that the attribution of maladaptive meanings to appearance-related UITs and the use of control strategies, such as body-focused checking and comparing behaviors, were key factors that contributed to the escalation of BDD morbid preoccupations. With regards to the dimensionality of illness-death UITs from normalcy to psychopathology, Arnáez et al. (2021) showed that there was a continuum of increasing frequency and disturbance caused by IAD-related intrusive cognitions from non-clinical to severe IAD. Illness-death UITs also have a significant emotional impact on non-clinical individuals (Pascual-Vera et al., 2017, 2019), and patients with IAD often develop maladaptive beliefs (i.e., overestimation of the likelihood of illness) and engage in counterproductive behaviors to manage these UITs (Arnáez et al., 2021; Muse et al., 2010). Recently, Pascual-Vera and Belloch (2022) showed that up to 72% of OCD patients experience OCD, BDD, and IAD-related UITs, causing a similar degree of disturbance, regardless of their content, and instigating a “*need to do something*”. In summary, these findings support the notion that UITs are not exclusive to clinical populations and that the misinterpretations of these thoughts by non-clinical subjects are similar to those observed in individuals with OCD, BDD, and IAD.

The objective of this study was to investigate whether emotional reactions, dysfunctional appraisals, and neutralizing behaviors observed in the progression from obsessional UITs to obsessions, parallel the transition from BDD- and IAD-related UITs to the clinical symptoms of these disorders. Obtaining data on this issue could have clinical implications as it would allow clinicians to obtain more accurate data about the relative weight of several maladaptive consequences for the different disorders in which UITs are involved. It could also allow establishing to what extent the different maladaptive consequences influence the vulnerability of the disorders to which the UITs are relevant, helping to increase the efficacy of treatments and/or to reduce the likelihood of relapse and residual symptoms. From this perspective, the main aim of the current research was to explore whether a main assumption of the cognitive-behavioral model of OCD, i.e., that the escalation of normal obsessional-related UITs to clinically significant obsessions depends on the misinterpretations, negative emotions, and counterproductive behaviors of those UITs, could also be applied to other UIT contents, particularly those associated with BDD and IAD. Moreover, as the three disorders are included within the same diagnostic group (WHO, 2024), with OCD considered the organizing or main disorder, we sought to investigate whether the same misinterpretations and behavioral consequences of having an obsessional-related UIT, also mediates the relationship between the frequency of UITs about appearance defects and about illness/death with measures of BDD and IAD symptoms, respectively.

## 2. Materials and Methods

### 2.1. Study Population and Procedure

A total of 625 adult volunteers participated in the study. Participants were non-clinical individuals from Ankara (Turkey), Buenos Aires (Argentina), Tel Aviv (Israel), and Valencia (Spain). Having diagnosed mental health problems in the past six months, undergoing psychological or pharmacological treatment (criterion a), or not being in the 18-to-65 age range (criterion b) were non-inclusion criteria. Participants who reported current mental health problems in the socio-demographic questionnaire (see Section 2.2) and/or did not meet the age eligibility criterion were not included in the data analysis. In total, 65 individuals were excluded: 55 based on criterion (a), and 11 based on criterion (b). Participants were recruited by the authors from several sources: undergraduate students who attended their lectures at the University, advertisements on the University Campus, and the web page of each research group, requesting voluntary participation in a study on values and beliefs about thoughts. Informed consent of the participants was obtained after the nature of the procedures had been fully explained. Those who explicitly agreed to participate and provided informed written consent were scheduled to attend an assessment session. In this session, the self-report instrument booklet was administered to groups of 25–35 individuals in the presence of one of the authors. The protocol was administered in writing in all cases. A standard data template was created to enter the same labels and categories across sites. The study design was approved by the ethical committee boards of each research site, in accordance with the latest version of the Declaration of Helsinki.

### 2.2. Instruments

*Socio-demographic data sheet*. The required data were the following: age, gender, years of education, marital status, and socio-economic level. Two additional questions about the participants’ current mental health status were also included: (1) Have you been diagnosed with a mental or behavioral disorder during the past six months, or are you currently receiving treatment for a mental or behavioral disorder? (2) If yes, could you please write the diagnosis and/or the treatment you are receiving for this mental or behavioral disorder?

*Questionnaire of Unpleasant Intrusive Thoughts* (QUIT; Pascual-Vera et al., 2017). The QUIT explores the transdiagnostic nature of UITs. It is a self-report questionnaire based on previous self-reports assessing the frequency of UITs with different contents: OCD-related (García-Soriano et al., 2011), BDD-related (Giraldo-O’Meara & Belloch, 2018, 2019), related to illness and death (Arnáez et al., 2021), and related to eating disorders (Belloch et al., 2016). Two pilot studies of the Spanish version of the QUIT were conducted with Spanish community samples (Pascual-Vera et al., 2017; Pascual-Vera & Belloch, 2018). Following these studies, the Spanish version of the QUIT was first translated into English, and then back-translated into Spanish by an accredited English–Spanish translator. The final English version of the QUIT was subsequently translated and back-translated into Hebrew and Turkish, following a standardized translation/back-translation protocol adopted by all authors.

The QUIT starts with a detailed definition of UITs and includes the different ways that the UITs could be experienced (i.e., as thoughts/doubts, images, impulses, or physical sensations). After the initial description, four separate sets of UITs are presented: OCD-related (12 items), BDD-related (9 items)*,* related to illness and death (10 items), and related to eating disorders (8 items). Respondents must evaluate each UIT on its frequency (from 0 = never to 6 = always) and the discomfort (from 0 = *not at all* to 4 = *extremely*) it causes when it occurs. In this study, only the OCD, BDD, and IAD intrusive thoughts were included. After completing each set of UITs, the respondent is asked to choose the most upsetting UIT they experienced during the past three months with a frequency of at least ≥1 (“*Rarely, once or twice*”) from the previous list. Next, the functional consequences associated with the most upsetting intrusion are evaluated through 14 items (from 0 = never/not at all to 4 = always/frequently) to assess the emotional distress experienced (2 items: anxiety, sadness), the interference (“*It interrupts what I’m doing; it distracts me*”), the associated egodystonicity (“*I find it unacceptable; it goes against what I want, or against my values and beliefs*”), the dysfunctional appraisals that the individual attaches to the intrusion (3 items: thought importance, the importance of thought control, and thought-action fusion), and the control and/or neutralizing strategies the person uses to manage the UIT (7 items: do nothing, stop thinking/thought suppression, self-punishment, repetitive overt/covert behaviors, reassurance, positive distraction, and avoidance). In the current study, only the strategies of thought suppression and overt/covert neutralizing behaviors were computed, as they include the following examples: “*organize, check, touch things or myself, wash, clean, pray, count, repeat an action, say a particular word or phrase, repeat a prayer, think opposite thought,* etc.”. The rationale behind this decision was to focus the study exclusively on those neutralizing strategies that are included in the current diagnostic criteria (i.e., ICD-11 and DSM-5-TR) of the three disorders of interest (OCD, BDD, and IAD), namely, repetitive behaviors or mental acts. The inclusion of thought suppression was based on its well-established relevance in OCD, as supported by a substantial amount of research and by its inclusion as a diagnostic criterion for the disorder. Given that OCD is the organizing disorder of the obsessive-compulsive spectrum group of disorders, in which BDD and IAD are included in the ICD-11 (WHO, 2024), we sought to explore the putative importance of thought suppression to manage and face up to the BDD and IAD-related UITs.

For the self-report questionnaire measures that follow, Spanish, Turkish and Hebrew published translations with established norms were used when available. In cases where the following measures were not available in the language of testing in a particular site, the same translation/back-translation protocol used for the QUIT was employed.

*Obsessive-Compulsive Inventory-Revised* (OCI-R; Foa et al., 2002). It is an 18-item self-report questionnaire that assesses distress associated with obsessive-compulsive symptoms. The questionnaire is answered on a 5-point Likert scale, ranging from 0 (not at all) to 4 (very much). The OCI-R measures six dimensions of OC symptoms: washing, checking, obsessing, hoarding, neutralizing, and ordering. Higher scores indicate a greater degree of distress associated with O-C symptoms. Previous data suggested that the OCI-R possesses good internal consistency for the total score and adequate test–retest reliability across samples (Foa et al., 2002). In our study, we used the total score (range from 0 to 72), and the internal consistency was excellent: *α* = 0.90.

*Body Dysmorphic Disorder Questionnaire* (BDDQ; Phillips et al., 1995). This is a brief screening instrument for BDD, according to the DSM-IV criteria, aimed at assessing respondents’ dysmorphic concerns and, if these concerns are experienced, the impairment they cause. The BDDQ can be completed either as a self-report or by an interviewer. In this study, it was administered as a self-report questionnaire, which demonstrates high sensitivity and specificity. It has been recommended for screening in psychiatric and cosmetic/surgery settings, as well as in general populations (e.g., Türk et al. 2023). The BDDQ includes 12 questions with various response formats (yes/no, open-ended, and multiple choice). The first two questions (yes/no response format) and the third (open response) are used as a screening tool for BDD, with a negative or blank answer to any of these first three questions instructing the respondent to stop completing the rest of the questionnaire. Taking this into account, only 82 participants completed the entire instrument. The total score ranges from 0 to 7 and is considered a score of distress and interference due to dysmorphic concerns. It has been calculated by adding the positive responses to: (a) the first two items (worries about physical appearance), plus (b) the four questions about the impact that appearance concerns have on life, and (c) the item on the amount of time spent daily thinking about appearance (at least 1–3 h or more per day). In this study, the internal consistency of this composite score was low (Cronbach’s alpha = 0.57).

*Whiteley Index* (WI; Pilowsky et al., 1984). This is one of the first dimensional measures to assess health anxiety. Its items are based on clinicians’ experiences of illness characteristics of severe health anxiety or hypochondriasis. It contains 14 True/False items, yielding a total score range of 0–14. It has demonstrated good internal consistency, stability and validity in individuals from the general population (e.g., Speckens et al., 1996). This study used the total score, and the internal consistency was excellent: *α* = 0.84.

### 2.3. Data Analyses

All statistical analyses were performed using IBM SPSS Statistics (version 22). An alpha level of 0.05 (two-tailed) was used for all statistical tests. Intercorrelations among all study measures were carried out by computing Pearson’s correlations. To analyze the mediating variables between the frequency of intrusions and symptoms of self-reports, parallel multiple mediation analyses were conducted using ‘model 4’ from the PROCESS macro for SPSS, version 2.15 (Hayes, 2017). Subsequently, three mediation models (one for each intrusion content separately) were tested: the frequency of the selected UIT (e.g., OCD-intrusions) was entered as the independent variable, the scores of self-reported symptoms (e.g., OCI-R total score) were entered as the dependent variable, and the associated maladaptive consequences were entered as the mediators. The mediators included in each model were emotional impact, interference, egodystonicity, dysfunctional appraisals, thought suppression, and overt/covert neutralizing behaviors. No covariates, such as age, gender or sample location, were included in the mediation analyses. Models were tested through the PROCESS macro for SPSS, and a bootstrapping approach was applied: mediation exists when a 95% CI of the indirect effect estimated from the bootstrap procedure excludes zero (Hayes, 2017). Ten thousand bootstrap samples and 95% bias-corrected CIs were used to evaluate the significance of the indirect effect, and in case it was significant, its effect size was measured as the ratio of indirect to total effect. The sample size was not estimated a priori using simulation methods. We followed Hayes’s (2017) recommendations regarding the use of bootstrapping and the minimum suggested sample size for complex models.

## 3. Results

### 3.1. Participant’s Characteristics and Preliminary Data

From the total sample (N = 625), 79.2% of participants were women, and their mean age was 27.98 years (*SD* = 11.75; range = 18–64 years). The highest proportion of women was in Turkey (*χ*^2^(3, N = 625) = 55.350, *p* < 0.001), and the Israeli and Argentinian participants were the oldest (*F*_3,624_ = 183.275, *p* < 0.0001). The demographic characteristics of participants by site are in Table 1.

From the total sample, up to 54.88% (*N* = 343) of the participants reported experiencing all three types of highly disturbing UITs included in the study within the past three months. Considering the three UIT types separately, 547 participants (87.52%) experienced a highly OCD-related disturbing thought in the past three months, 460 (73.6%) had a BDD-related intrusion, and 437 (69.92%) reported an IAD-related thought. The mean frequency of the UITs and the associated maladaptive consequences are presented in Table 2. Participants reported experiencing OCD-related and BDD-related intrusions between once or twice a month to weekly, whereas IAD-related intrusions were experienced a few times a year. Overall, the maladaptive consequences ranged from “occasionally/very little” to “sometimes/somewhat” when participants experienced the most disturbing UITs.

### 3.2. Associations Between the Frequency of the Most Upsetting UITs, Their Maladaptive Consequences, and Symptom Measures of OCD, BDD, and IAD

Bivariate correlations showed that all study variables were interrelated. As shown in Table 3, the frequency of each UIT content correlated with its corresponding clinical measure.

Moreover, the frequency of the selected UIT and symptom measures of each disorder correlated with its corresponding maladaptive consequences: the frequency of OCD-related UITs and the OCI-R score correlated with the maladaptive consequences associated with the intrusion. Overall, the largest coefficients were found between the OCI-R and the dysfunctional appraisals (*r* = 0.513, *p* < 0.001), while the smallest, and only marginal coefficients, were between the frequency of the OCD-related UITs and thought suppression (*r* = 0.09, *p <* 0.05). All maladaptive consequences were related to each other, ranging from *r* = 0.192 to *r* = 0.504 (all *p*’s ≤ 0.01).

The frequency of the BDD-related intrusive thoughts correlated with their respective negative consequences, except for thought suppression. Nonetheless, even though all maladaptive consequences were interrelated (*r* range from 0.19 to 0.59; all *p*’s ≤ 0.01), the score on the BDDQ was only associated with the emotional impact (*r* = 0.25, *p* < 0.05) and the interference (*r* = 0.33, *p* < 0.01) caused by the intrusive thought. As explained when describing the BDDQ, these analyses were based on the scores of the 82 participants who completed the BDDQ.

Finally, the frequency of IAD-related intrusive thoughts and the score on the Whiteley Index correlated with the maladaptive consequences of the IAD intrusions. The most significant coefficients were between the following pairs of correlations: the frequency of IAD-related UITs and the dysfunctional appraisals (*r* = 0.331; *p* ≤ 0.01), and the WI and the dysfunctional appraisals (*r* = 0.288; *p* ≤ 0.01). Similarly, the lowest coefficients were found between the frequency of IAD-related UITs and egodystonicity (*r* = 0.140; *p* ≤ 0.01) and between the WI and egodystonicity (*r* = 0.143; *p* ≤ 0.01). Moreover, all maladaptive consequences were interrelated, ranging from *r =* 0.265 to *r* = 0.692 (all *p’s* ≤ 0.01).

### 3.3. Mediating Role of Maladaptive Consequences on the Relationship Between the Frequency of the Most Disturbing UIT and Symptoms of OCD, BDD, and IAD

**Figure 1:**
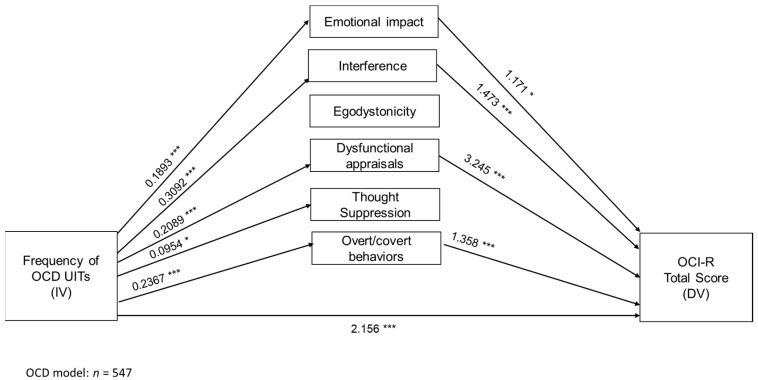
Direct effects between the frequency of the OCD-related intrusive thought, the maladaptive consequences of said thought, and the OCI-R total score. Data are significant unstandardized regression coefficients. * *p* ≤ 0.05; *** *p* ≤ 0.001.

**Figure 2:**
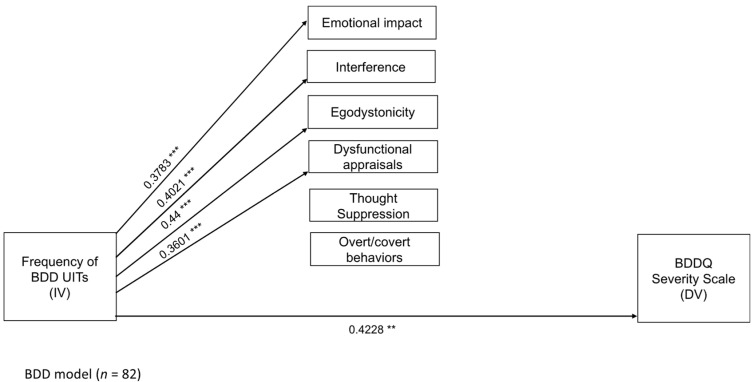
Direct effects between the frequency of the BDD-related intrusive thought, the maladaptive consequences of said thought, and the BDDQ. Data are significant unstandardized regression coefficients. ** *p* ≤ 0.01; *** *p* ≤ 0.001.

**Figure 3:**
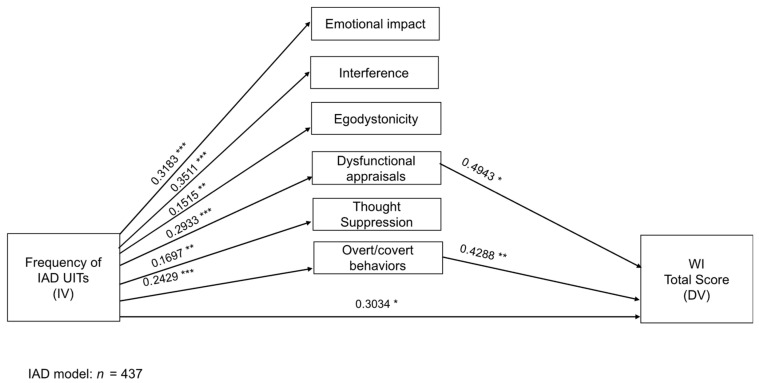
Direct effects between the frequency of IAD UITs, the maladaptive consequences of IAD intrusions, and the WI. Data are significant unstandardized regression coefficients. * *p* ≤ 0.05; ** *p* ≤ 0.01; *** *p* ≤ 0.001.

Table 4 and Table 5 present the significant direct and indirect effects of the three mediational models. Non-significant effects of each model are reported in the Supplementary Material. Figure 1, Figure 2 and Figure 3 display the significant unstandardized regression coefficients for the OCD, BDD and IAD models, respectively.

Regarding the OCD model, results show that the frequency of the OCD-related intrusive thought positively predicted the scores on their maladaptive consequences, except for the egodystonicity caused by the thought. The scores for emotional impact, interference, dysfunctional appraisals, and neutralizing strategies were predictive of the OCI-R total score. Moreover, the frequency of the OCD-related intrusive thought predicted the score of the OCI-R (see Figure 1 and Table 4). The overall model was significant (*F*_7,539_ = 52.9424, *p* ≤ 0.001), explaining 40.74% of the variance in OCI-R. As shown in Table 5, the indirect effect of the frequency of OCD-related UITs through emotional impact, interference, dysfunctional appraisals, and neutralizing behaviors on the total OCI-R was significant, suggesting that these maladaptive consequences mediate the path.

Results of the BDD model are presented in Figure 2 and Table 4. As shown, the frequency of the BDD-related intrusive thoughts predicted the scores on the emotional impact, interference, egodistonicity, and dysfunctional appraisals attached to the appearance-defect intrusion. The frequency of this UIT also predicted the scores on the BDDQ-distress and interference (direct effects). The model explained 22.98% of the BDDQ variance (*F*_7,74_ = 3.1534, *p* ≤ 0.01). Nonetheless, no indirect effects of the frequency of the BDD-related intrusive thoughts on the BDDQ scores through maladaptive consequences were observed. Thus, none of the maladaptive consequences mediated the relationship between the frequency of BDD-related UITs and BDD symptom severity. Full details of the non-significant mediation paths are provided in the Supplementary Materials.

Regarding the IAD model, results showed that the frequency of the IAD-related intrusive thoughts positively predicted the scores on their negative consequences (see Table 4). Moreover, both the frequency of this IAD thought as well as the attached dysfunctional appraisals and neutralizing behaviors were predictive of the WI scores (direct effects). The overall model was significant (*F*_7,429_ = 6.7986, *p* ≤ 0.001), although it explained only 9.99% of the variance in the WI total score. Nonetheless, the indirect effect of the frequency of IAD-related intrusive thoughts through dysfunctional appraisals and neutralizing behaviors on the WI scores was significant (see Table 5). Figure 3 shows the results of the multiple parallel mediation IAD model.

## 4. Discussion

Cognitive-behavioral theories suggest that the progression from universal and common unwanted intrusive thoughts to clinically significant symptoms of OCD, BDD and IAD depends on how individuals interpret these UITs and how they respond to and manage them (e.g., Clark, 2004; Marcus et al., 2007; Wilhelm et al., 2013). This study aimed to investigate how maladaptive consequences resulting from unwanted intrusive thoughts mediate the clinical symptoms of the three disorders mentioned above. These disorders are currently clustered together in the ICD-11 (WHO, 2024), reflecting the proposals that highlight the similarities in terms of their phenomenology and functionality (Abramowitz & Braddock, 2006; Abramowitz & Jacoby, 2015).

In our current study, more than half of the non-clinical participants reported experiencing three types or contents of disturbing UITs. This data is consistent with the grouping of OCD, BDD, and IAD under the same umbrella of disorders that share the experience of intrusive, repetitive, and uncontrollable thoughts.

The frequency of intrusions with contents specific and/or relevant to each disorder directly predicted the scores on the clinical measures of its respective disorder (e.g., OCD-UITs-OCI-R), supporting the role of highly disturbing UITs in the genesis and/or maintenance of OCD, BDD, and IAD symptoms. The fact that the frequency of the UIT selected by the participants as the most disturbing predicted (i.e., direct effects) the vast majority of the maladaptive consequences (i.e., emotions, appraisals and neutralizing strategies), specifically in the OCD and IAD models, provide support to this assumption, suggesting that a functional link exists between the experience of an unwanted and recent obsessional and illness-related UIT, and the maladaptive consequences used to alleviate the discomfort it causes (Pascual-Vera et al., 2022; Pascual-Vera & Belloch, 2018, 2022).

Results of the indirect effects support the critical role of cognitive and behavioral dimensions in the genesis, persistence, and treatment of OCD (e.g., Abramowitz et al., 2017; Clark, 2019) given that the emotional impact, interference, dysfunctional appraisals, and overt/covert behaviors experienced after an OCD-related intrusive thought specifically mediated the relationship between the frequency of this OCD-UIT and the OCI-R scores (both direct and indirect effects). Overall, findings support cognitive-behavioral assumptions on OCD, although some unexpected results deserve additional comment. The absence of significant mediation of thought suppression suggests that this type of neutralizing strategy is highly dysfunctional and represents a shift from normalcy to OCD. This has been demonstrated in studies on thought suppression in individuals with OCD as well as non-clinical samples when confronted with unwanted intrusive thoughts (i.e., Purdon & Clark, 2001; Tolin et al., 2002; Trinder & Salkovskis, 1994). The lack of statistically significant results regarding egodystonicity could be due, first, by the low frequency of the obsessional UITs, and second, by the possibility that the content of the intrusive thought was not itself egodystonic, as it is observed in clinical settings with some obsessions related to ordering or cleaning that individuals do not evaluate as contrary to their self-view and values. In fact, the wording of the item is as follows “*I find it unacceptable; it goes against what I want, or against my values and beliefs*”.

The absence of indirect effects in the frequency of BDD intrusions on BDD symptoms through the maladaptive consequences of having intrusive thoughts related to appearance defects does not support the idea that guided this research. This could be due to both methodological limitations and theoretical reasons. The low number of individuals who completed the BDDQ (*n* = 82), the low internal consistency of the total score, and the limitations of this questionnaire as a symptom measure of BDD could explain, at least in part, these results. The BDDQ-score is a measure of the distress and interference caused by intrusive thoughts about appearance defects, but only in respondents at risk of BDD, which means that 82 participants (18.76% of the individuals who reported having had a BDD-related intrusive thought in the last three months) were highly vulnerable to the disorder. The high correlation coefficient between the BDDQ score and the frequency of the BDD-related UIT supports that assumption and endorses the use of the BDDQ as a screening tool for BDD in non-general populations, psychiatric services, and surgery/cosmetic settings (Türk et al., 2023). Therefore, other potential negative consequences, not assessed by the QUIT but functionally related to intrusive thoughts about appearance defects, might mediate BDD psychopathology. Experts in BDD (e.g., Wilhelm et al., 2013; Veale, 2004) suggest that individuals with BDD respond to appearance-related UITs by making negative appraisals about their appearance, which include self-judgments and perceived judgments of others. Therefore, these appraisals are not about the intrusion itself but about the perceived defect, which suggests that subjects are in agreement with the thought and, for this reason, it is not evaluated negatively. Moreover, similar to individuals with eating disorders, individuals vulnerable to BDD might consider the UIT as a suggestion to take action to correct, conceal, or reduce their perceived appearance flaw. The absence of a correlation between the frequency of the BDD-related UIT and the thought suppression strategy supports this argument. In their study about appearance defect UITs, Giraldo-O’Meara and Belloch (2018) found that individuals with BDD used more social control strategies, such as avoidance, concealment, and/or comparing, than both the general population and individuals vulnerable to BDD. The QUIT does not assess two of these important social control strategies, concealment and comparison, which is a limitation of the questionnaire. However, above all, it is more likely that behind the results are the sample characteristics, predominantly women and young, two factors that influence the low insight and low egodystonicity of intrusive thoughts about appearance defects typically found in BDD (Phillips et al., 2007). Anyway, our findings are far from supporting the idea that the same misinterpretations and behaviors involved in the OCD are also involved in the escalation of intrusive thoughts about appearance defects into symptoms of BDD.

In the illness anxiety disorder model, the dysfunctional appraisals and overt/covert behaviors mediated the relationship between the intrusive thought and IAD symptoms, which support the cognitive-behavioral model of this disorder and their similarities with OCD, specially taking into account that the misinterpretations and behaviors assessed by the QUIT were those typically endorsed by individuals with OCD. The role of faulty appraisals in IAD coincides with Arnáez et al. (2021), who reported that a combination of dysfunctional appraisals (e.g., overestimation of threat, thought-action-fusion) mediated the relationship between the frequency of illness UITs and health anxiety symptoms in both the general population and patients with IAD. Last, our findings suggest that overt/covert behaviors may be common processes linked to obsessional and illness-related UITs, as revealed by prior findings in OCD and IAD patients and in the general population (Pascual-Vera & Belloch, 2018). Nonetheless, the low variance explained by the model calls into question its explanatory power and the generalizability of results. One possible explanation is the low frequency of UITs related to illness and death reported by participants, which may be attributable to their relatively young average age (approximately 30 years). This interpretation aligns with findings by Pascual-Vera et al. (2017, 2019), who observed that, in the general population, illness-related UITs were infrequent but highly distressing. These results could suggest that, in the context of the IAD model, it may be more informative to examine the level of distress caused by illness-related UITs rather than their frequency, at least in younger non-clinical populations. Additionally, the weak correlation between the frequency of IAD-related UITs and the WI may further account for the limited explanatory power observed.

This study has several limitations that should be mentioned. First, the study design is cross-sectional, which limits the possibility of drawing causal inferences. Although there are theoretical reasons supporting the proposed order of mediators in the mediation models, longitudinal studies are needed to confirm these findings and establish causal assumptions. Additionally, no covariates were included in the mediation analyses, which prevents us from ascertaining the putative influence of culture, age or gender on the results. The fact that our participants were mainly young women from the general population limits again the generalizability of the results—particularly to male populations and individuals over the age of thirty. Although using non-clinical samples is common to examine dimensional variables such as UITs, it is important to confirm the data obtained with clinical participants. The low frequency of the UITs experienced by most participants limits the possibility of interference in daily life and its egodystonicity and, consequently, does not clearly instigate the need to keep the intrusive thought under control. Another limitation was the administration of the BDDQ as a self-report to assess BDD symptoms, especially given the low reliability scores observed in our sample. As mentioned in the instruments section, the BDDQ comprises a first screening part (the first three items) and a second severity and distress score (the rest of the items) that must only be completed after giving positive answers to the first part. These screening items ask about physical appearance problems and ED-concerns (e.g., thinness; item 4), which are also highly prevalent among BDD samples. Given these limitations and the results obtained in the BDD model, future studies should incorporate additional self-report instruments to assess symptoms, cognitions and behaviors related to BDD more comprehensively.

Despite these limitations, the results of this study have some implications for clinical practice. Previous research has supported the notion that the experience of distressing, unwanted intrusive thoughts constitutes a transdiagnostic risk factor across different cultural and social contexts, as well as among individuals diagnosed with OCD (Pascual-Vera et al., 2019, 2022). Consistent with this, the current findings also indicate that UITs predict symptoms of OCD, BDD, and IAD. Assessment and case formulation in these disorders should provide information about the clinical significance of UITs with various contents and themes, and not just those typically specific to each disorder, as experiencing UITs may be a relevant indicator of vulnerability. The findings support the cognitive aspects of OCD and, at least partially, those of IAD, which may allow the development of transdiagnostic treatment components based on these disorders, specifically targeting the dysfunctional appraisals and overt and covert behaviors associated with UITs. The data also supports the contribution of the frequency of BDD UITs to BDD symptoms, although future studies should include more specific BDD variables (i.e., misinterpretations and behaviors) to examine the role of the maladaptive consequences of appearance-related UITs in the disorder.

## Figures and Tables

**Table 1:** Participants’ characteristics by site.

Country	*n*	Age *M* (*SD*)	Gender % Female
Spain	92	28.3 (11.58) ^a^	71.7 ^a^
Israel	78	40.35 (12.8) ^c^	50 ^b^
Turkey	346	21.52 (4.52) ^b^	86.1 ^c^
Argentina	109	39.36 (11.3) ^c^	84.4 ^ac^
Overall	625	27.98 [11.75]	79.2

*Note.* Values that share the same superscript letter were not significantly different from each other. Differing superscript letters indicate post-hoc between-groups differences (Dunnett and Bonferroni corrections for Mean (*SD*) and %, respectively).

**Table 2:** Frequency and maladaptive consequences of OCD, BDD and IAD intrusive thoughts.

	Unwanted Intrusive Thoughts			
	OCD-Related	BDD-Related	IAD-Related	Range
Frequency most disturbing	3.34 (1.42)	3.1 (1.42)	2.42 (1.16)	0–6
Emotional impact	1.45 (1.08)	1.58 (1.07)	2.10 (1.19)	0–4
Interference	1.88 (1.24)	1.43 (1.21)	1.65 (1.32)	0–4
Egodystonicity	1.02 (1.35)	0.94 (1.21)	0.86 (1.24)	0–4
Dysfunctional Appraisals	1.41 (0.93)	1.34 (1)	1.47 (1.04)	0–4
Thought Suppression	1.84 (1.37)	1.68 (1.32)	1.88 (1.38)	0–4
Overt/covert behaviors	1.58 (1.43)	1.39 (1.39)	1.38 (1.37)	0–4

*Note.* Data are mean (SD).

**Table 3:** Correlations among the frequency of the most disturbing OCD, BDD, and IAD-related unwanted intrusive thoughts and symptom measures.

Frequency of the Most Upsetting UIT	Symptom Self-Reports			
		OCI-R Total	BDDQ-Severity	Whiteley Index
OCD-related	*r*	0.439 **	0.162	0.136 **
	*n*	560	96	557
BDD-related	*r*	0.288 **	0.447 **	0.132 **
	*n*	465	82	463
IAD-related	*r*	0.248 **	0.296 *	0.181 **
	*n*	443	73	441

*Note.* * *p* < 0.05; ** *p* < 0.01; OCI-R: Obsessive-Compulsive Inventory-Revised; BDDQ: Body Dysmorphic Disorder Questionnaire.

**Table 4:** Significant direct effects of the frequency of UITs through the maladaptive consequences on OCD, BDD and IAD symptoms.

	SE	95% CI	β	R^2^
*OCD model (IV: Frequency of OCD-UIT; DV: OCI-R)*				
Frequency → Emotional impact	0.0312	[0.1279, 0.2506]	0.2488	0.06
Frequency → Interference	0.0346	[0.2412, 0.3773]	0.3540	0.12
Frequency → Dysfunctional appraisals	0.0265	[0.1568, 0.2610]	0.3189	0.10
Frequency → Thought Suppression	0.0408	[0.0151, 0.1756]	0.0988	0.00
Frequency → Overt/covert behaviors	0.0419	[0.1544, 0.3189]	0.2350	0.05
Emotional impact → OCI-R	0.51	[0.1694, 2.173]	0.1009	
Interference → OCI-R	0.4192	[0.6502, 2.297]	0.1457	
Dysfunctional appraisals → OCI-R	0.5993	[2.068, 4.423]	0.2408	
Overt/covert behaviors → OCI-R	0.3246	[0.7212, 1.996]	0.1549	
Total direct effect	0.3260	[1.515, 2.796]	0.2443	
*BDD model (IV: Frequency of BDD-UIT; DV:* *BDDQ-Severity Scale)*				
Frequency → Emotional impact	0.0943	[0.1907, 0.5659]	0.5020	0.16
Frequency → Interference	0.1028	[0.1976, 0.6067]	0.4718	0.16
Frequency → Egodystonicity	0.1148	[0.2115, 0.6686]	0.5163	0.15
Frequency → Dysfunctional appraisals	0.0934	[0.1741, 0.5461]	0.5113	0.15
Total direct effect	0.1465	[0.1309, 0.7147]	0.4056	
*IAD model (IV: Frequency of IAD-UIT; DV: WI)*				
Frequency → Emotional impact	0.0446	[0.2306, 0.4060]	0.3102	0.10
Frequency → Interference	0.0513	[0.2502, 0.4520]	0.3085	0.09
Frequency → Egodystonicity	0.0514	[0.0505, 0.2526]	0.1417	0.01
Frequency → Dysfunctional appraisals	0.04	[0.2146, 0.3719]	0.3271	0.10
Frequency → Thought Suppression	0.0552	[0.0611, 0.2782]	0.1426	0.02
Frequency → Overt/covert behaviors	0.0550	[0.1347, 0.3511]	0.2056	0.04
Dysfunctional appraisals → WI	0.2141	[0.0736, 0.9151]	0.1420	
Overt/covert behaviors → WI	0.1451	[0.1436, 0.7141]	0.1622	
Total direct effect	0.1496	[0.0093, 0.5975]	0.0972	

*Note:* Data are standard errors (SEs), confidence intervals (CIs), standardized coefficients (β) and explained variance (R^2^) of the parallel multiple mediation analysis of the OCD, BDD and IAD models.

**Table 5:** Indirect effects of the frequency of UITs through the maladaptive consequences on OCD and IAD symptoms.

Indirect Effects	Coefficients	SE	95% CI	β
*OCD model (IV: Frequency of OCD-UIT; DV: OCI-R)*				
Total effect				0.4330
Total indirect effect	1.665	0.2354	[1.220, 2.144]	0.1886
Emotional impact	0.2217	0.1147	[0.0095, 0.4611]	0.0251
Interference	0.4557	0.1379	[0.1973, 0.7448]	0.0516
Dysfunctional appraisals	0.6780	0.1626	[0.3878, 1.0258]	0.0768
Overt/covert behaviors	0.3216	0.1002	[0.1456, 0.5344]	0.0364
*IAD model (IV: Frequency of IAD-UIT; DV: WI)*				
Total effect				0.1724
Total indirect effect	0.2349	0.0773	[0.0988, 0.4022]	0.0752
Dysfunctional appraisals	0.1450	0.0674	[0.0255, 0.2927]	0.0464
Overt/covert behaviors	0.1042	0.0461	[0.0290, 0.2045]	0.0333

*Note:* Data are unstandardized coefficients, standard errors (SEs), confidence intervals (CIs) and standardized coefficients (β) of the parallel multiple mediation analysis of the OCD and IAD models.

## Data Availability

The data that support the findings of this study are available from the first and corresponding authors (B.P.-V. and A.B., respectively) upon reasonable request.

## Supplementary Materials

The following supporting information can be downloaded at: https://www.mdpi.com/article/10.3390/ejihpe15070135/s1, Table S1: Non-significant effects (direct and indirect) of the frequency of UITs through the maladaptive consequences on OCD, BDD and IAD symptoms.

## References

[B1-ejihpe-15-00135] Abramowitz J. S., Braddock A. E. (2006). Hypochondriasis: Conceptualization, treatment, and relationship to obsessive compulsive disorder. Psychiatric Clinics of North America.

[B2-ejihpe-15-00135] Abramowitz J. S., Jacoby R. J. (2015). Obsessive-compulsive and related disorders: A critical review of the new diagnostic class. Annual Review of Clinical Psychology.

[B3-ejihpe-15-00135] Abramowitz J. S., McKay D., Storch E. A. (2017). The Wiley handbook of obsessive-compulsive disorders.

[B4-ejihpe-15-00135] Abramowitz J. S., Taylor S., McKay D. (2009). Obsessive-compulsive disorder. The Lancet.

[B5-ejihpe-15-00135] American Psychiatric Association (APA) (2022). Diagnostic and statistical manual of mental disorders.

[B6-ejihpe-15-00135] Arnáez S., García-Soriano G., López-Santiago J., Belloch A. (2021). Illness-related intrusive thoughts and Illness Anxiety Disorder. Psychology and Psychotherapy: Theory, Research and Practice.

[B7-ejihpe-15-00135] Belloch A., Roncero M., Perpiñá C. (2016). Obsessional and eating disorder-related intrusive thoughts: Differences and similarities within and between individuals vulnerable to OCD or to EDs. European Eating Disorders Review.

[B8-ejihpe-15-00135] Berry L., Laskey B. (2012). A review of obsessive intrusive thoughts in the general population. Journal of Obsessive-Compulsive and Related Disorders.

[B9-ejihpe-15-00135] Clark D. A. (2004). Cognitive-behavioral therapy for OCD.

[B10-ejihpe-15-00135] Clark D. A. (2005). Intrusive thoughts in clinical disorders: Theory, research, and treatment.

[B11-ejihpe-15-00135] Clark D. A. (2019). Cognitive-behavioral therapy for OCD and its subtypes.

[B12-ejihpe-15-00135] Clark D. A., O’Connor K., Clark D. A. (2005). Thinking is believing. Ego-dystonic intrusive thoughts in obsessive-compulsive disorder. Intrusive thoughts in clinical disorders.

[B13-ejihpe-15-00135] Clark D. A., Purdon C., Byers E. S. (2000). Appraisal and control of sexual and non-sexual intrusive thoughts in university students. Behaviour Research and Therapy.

[B14-ejihpe-15-00135] Eisen J. L., Phillips K. A., Coles M. E., Rasmussen S. A. (2004). Insight in obsessive compulsive disorder and body dysmorphic disorder. Comprehensive Psychiatry.

[B15-ejihpe-15-00135] Fang A., Wilhelm S. (2015). Clinical features, cognitive biases, and treatment of body dysmorphic disorder. Annual Review of Clinical Psychology.

[B16-ejihpe-15-00135] Fergus T. A. (2013). Repetitive thought and health anxiety: Tests of specificity. Journal of Psychopathology and Behavioral Assessment.

[B17-ejihpe-15-00135] Foa E. B., Huppert J. D., Leiberg S., Langner R., Kichic R., Hajcak G., Salkovskis P. M. (2002). The obsessive-compulsive inventory: Development and validation of a short version. Psychological Assessment.

[B18-ejihpe-15-00135] Frare F., Perugi G., Ruffolo G., Toni C. (2004). Obsessive-compulsive disorder and body dysmorphic disorder: A comparison of clinical features. European Psychiatry.

[B19-ejihpe-15-00135] García-Soriano G., Belloch A., Morillo C., Clark D. A. (2011). Symptom dimensions in obsessive-compulsive disorder: From normal cognitive intrusions to clinical obsessions. Journal of Anxiety Disorders.

[B20-ejihpe-15-00135] Giraldo-O’Meara M., Belloch A. (2018). Escalation from normal appearance related intrusive cognitions to clinical preoccupations in body dysmorphic disorder: A cross-sectional study. Psychiatry Research.

[B21-ejihpe-15-00135] Giraldo-O’Meara M., Belloch A. (2019). The appearance intrusions questionnaire: A self-report questionnaire to assess the universality and intrusiveness of preoccupations about appearance defects. European Journal of Psychological Assessment.

[B22-ejihpe-15-00135] Harvey A. G., Watkins E., Mansell W., Shafran R. (2004). Cognitive behavioural processes across psychological disorders: A transdiagnostic approach to research and treatment.

[B23-ejihpe-15-00135] Hayes A. (2017). Introduction to mediation, moderation, and conditional process analysis: A regression-based approach.

[B24-ejihpe-15-00135] Marcus D. K., Gurley J. R., Marchi M. M., Bauer C. (2007). Cognitive and perceptual variables in hypochondriasis and health anxiety: A systematic review. Clinical Psychology Review.

[B25-ejihpe-15-00135] Muse K., McManus F., Hackmann A., Williams M., Williams M. (2010). Intrusive imagery in severe health anxiety: Prevalence, nature and links with memories and maintenance cycles. Behaviour Research and Therapy.

[B26-ejihpe-15-00135] Obsessive Compulsive Cognitions Working Group (OCCWG) (2001). Development and initial validation of the Obsessive Beliefs Questionnaire and the Interpretation of Intrusions Inventory. Behaviour Research and Therapy.

[B27-ejihpe-15-00135] Obsessive Compulsive Cognitions Working Group (OCCWG) (2003). Psychometric validation of the obsessive beliefs questionnaire and the interpretation of intrusions inventory: Part I. Behaviour Research and Therapy.

[B28-ejihpe-15-00135] Osman S., Cooper M., Hackmann A., Veale D. (2004). Spontaneously occurring images and early memories in people with body dysmorphic disorder. Memory.

[B29-ejihpe-15-00135] Pascual-Vera B., Akin B., Belloch A., Bottesi G., Clark D. A., Doron G., Fernández-Alvarez H., Ghisi M., Gómez B., Inozu M., Jiménez-Ros M., Moulding R., Ruiz M. A., Shams G., Sica C. (2019). The cross-cultural and transdiagnostic nature of unwanted mental intrusions. International Journal of Clinical and Health Psychology.

[B30-ejihpe-15-00135] Pascual-Vera B., Akin B., Belloch A., Bottesi G., Clark D. A., Doron G., Fernández-Alvarez H., Ghisi M., Gómez B., Inozu M., Jiménez-Ros M., Moulding R., Ruiz M. A., Shams G., Sica C. (2022). Maladaptive consequences of mental intrusions with obsessive, dysmorphic, hypochondriac, and eating-disorders related contents: Cross-cultural differences. International Journal of Clinical and Health Psychology.

[B31-ejihpe-15-00135] Pascual-Vera B., Belloch A. (2018). Functional links of obsessive, dysmorphic, hypochondriac, and eating-disorders related mental intrusions. International Journal of Clinical and Health Psychology.

[B32-ejihpe-15-00135] Pascual-Vera B., Belloch A. (2022). Dysmorphic and illness anxiety-related unwanted intrusive thoughts in individuals with obsessive-compulsive disorder. Clinical Psychology and Psychotherapy.

[B33-ejihpe-15-00135] Pascual-Vera B., Roncero M., Belloch A. (2017). Are unwanted mental intrusions a transdiagnostic variable?. Psicothema.

[B34-ejihpe-15-00135] Phillips K. A., Atala K. D., Pope H. G., American Psychiatric Association (1995). Diagnostic instruments for body dysmorphic disorder. New research program and abstracts: 148th meeting of the American Psychiatric Association.

[B35-ejihpe-15-00135] Phillips K. A., Didie E. R., Menard W., Pagano M. E., Fay C., Weisberg R. B. (2006). Clinical features of body dysmorphic disorder in adolescents and adults. Psychiatry Research.

[B36-ejihpe-15-00135] Phillips K. A., Pinto A., William-Menard B. A., Eisen J. L., Mancebo M., Rasmussen S. A. (2007). Obsessive-compulsive disorder versus body dysmorphic disorder: A comparison study of two possibly related disorders. Depression and Anxiety.

[B37-ejihpe-15-00135] Pilowsky I., Spence N., Cobb J., Katsikitis M. (1984). The illness behavior questionnaire as an aid to clinical assessment. General Hospital Psychiatry.

[B38-ejihpe-15-00135] Purdon C., Clark D. A. (2001). Suppression of obsession-like thoughts in nonclinical individuals: Impact on thought frequency, appraisal, and mood state. Behaviour Research and Therapy.

[B39-ejihpe-15-00135] Rachman S. (1997). A cognitive theory of obsessions. Behaviour Research and Therapy.

[B40-ejihpe-15-00135] Rowa K., Purdon C. (2003). Why are certain intrusive thoughts more upsetting than others?. Behavioural and Cognitive Psychotherapy.

[B41-ejihpe-15-00135] Salkovskis P. M. (1985). Obsessional-compulsive problems: A cognitive-behavioral analysis. Behaviour Research and Therapy.

[B42-ejihpe-15-00135] Solem S., Borgejordet S., Haseth S., Hansen B., Håland Å., Bailey R. (2015). Symptoms of health anxiety in obsessive-compulsive disorder: Relationship with treatment outcome and metacognition. Journal of Obsessive-Compulsive and Related Disorders.

[B43-ejihpe-15-00135] Speckens A. E., Spinhoven P., Sloekers P. P., Bolk J. H., van Hemert A. M. (1996). A validation study of the whitely index, the illness attitude scales, and the somatosensory amplification scale in general medical and general practice patients. Journal of Psychosomatic Research.

[B44-ejihpe-15-00135] Striegel-Moore R. H., Bulik C. M. (2007). Risk factors for eating disorders. American Psychologist.

[B45-ejihpe-15-00135] Tolin D. F., Abramowitz J. S., Hamlin C., Foa E. B., Synodi D. S. (2002). Attributions for thought suppression failure in obsessive-compulsive disorder. Cognitive Therapy and Research.

[B46-ejihpe-15-00135] Trinder H., Salkovskis P. M. (1994). Personally relevant intrusions outside the laboratory: Long term suppression increases intrusion. Behaviour Research and Therapy.

[B47-ejihpe-15-00135] Türk C. B., Maymone M. B., Kroumpouzos G. (2023). Body dysmorphic disorder: A critical appraisal of diagnostic, screening, and assessment tools. Clinical Dermatology.

[B48-ejihpe-15-00135] Veale D. (2004). Advances in a cognitive behavioural model of body dysmorphic disorder. Body Image.

[B49-ejihpe-15-00135] Veale D., Neziroglu F. (2010). Body dysmorphic disorder: A treatment manual.

[B50-ejihpe-15-00135] Wells A., Hackmann A. (1993). Imagery and core beliefs in health anxiety: Content and origins. Behavioural and Cognitive Psychotherapy.

[B51-ejihpe-15-00135] Wilhelm S., Neziroglu F., Frost R. O., Steketee G. (2002). Cognitive theory of body dysmorphic disorder. Cognitive approaches to obsessions and compulsions: Theory, assessment and treatment.

[B52-ejihpe-15-00135] Wilhelm S., Phillips K. A., Steketee G. (2013). Cognitive-behavioral therapy for body dysmorphic disorder: A treatment manual.

[B53-ejihpe-15-00135] World Health Organization (WHO) (2024). Clinical descriptions and diagnostic requirements for ICD-11 mental, behavioural and neurodevelopmental disorders.

