# Supplementary material for: The Mediating Role of Misinterpretations and Neutralizing Responses to Unwanted Intrusive Thoughts in Obsessive-Compulsive Spectrum Disorders
Source: Eur J Investig Health Psychol Educ. 2025 Jul 15;15(7):135. doi: 10.3390/ejihpe15070135 (PMC12294069; doi:10.3390/ejihpe15070135)
Supplement: Supplementary file 1 [file ejihpe-15-00135-s001.zip › ejihpe-3595841-supplementary.pdf]

SUPPLEMENTARY MATERIAL

Table S1. Non-significant effects (direct and indirect) of the frequency of UITs through the maladaptive consequences on OCD, BDD and IAD symptoms.

	**Coefficients**	**SE**	**95% CI**	*p*	R^2
OCD model (IV: Frequency of OCD-UIT; DV: OCI-R)					
**Direct effects**					
Frequency→ Egodystonicity	-0.0156	0.0407	[-0.0955, 0.0643]	0.7012	0.0003
Egodystonicity→OCI-R	0.4989	0.3538	[-0.1961, 1.1939]	0.1591	
**Indirect effects**					
Egodystonicity	-0.0078	0.0267	[-0.0681, 0.0448]		
BDD model (IV: Frequency of BDD-UIT; DV: BDDQ-Severity Scale)					
**Direct effects**					
Frequency→Thought Suppression	0.0388	0.1291	[-0.2181,0.2956]	0.7646	0.0011
Frequency→Overt/covert behaviors	0.0837	0.1356	[-0.1861,0.3535]	0.5388	0.0047
Emotional impact→ BDDQ	0.0617	0.1847	[-0.3063,0.4297]	0.7392	
Interference → BDDQ	0.2318	0.1696	[-0.1062,0.5699]	0.1760	
Egodystonicity→ BDDQ	-0.1895	0.1441	[-0.4767,0.0977]	0.1926	
Dysfunctional appraisals→ BDDQ	0.1471	0.2390	[-0.3291,0.6233]	0.5401	
Thought Suppression → BDDQ	-0.1837	0.1424	[-0.4675,0.1001]	0.2011	
Overt/covert behaviors→ BDDQ	-0.1228	0.1121	[-0.3461,0.1006]	0.2770	
**Indirect effects**					
Total indirect effect	0.0687	0.0920	[-0.1100,0.2543]		
Emotional impact	0.0233	0.0640	[-0.0874,0.1722]		
Interference	0.0932	0.0713	[-0.0421,0.2418]		
Egodystonicity	-0.0834	0.0715	[-0.2394 ,0.0495]		
Dysfunctional appraisals	0.0530	0.0930	[-0.1348,0.2457]		
Thought Suppression	-0.0071	0.0324	[-0.0848,0.0532]		
Overt/covert behaviors	-0.0103	0.0241	[-0.0762,0.0204]		
IAD model IAD model (IV: Frequency of IAD-UIT; DV: WI)					
**Direct effects**					
Emotional impact→ WI	-0.1707	0.2027	[-0.5691,0.2277]	0.4002	
Interference → WI	0.1202	0.1764	[-0.2266,0.4669]	0.4961	
Egodystonicity→ WI	0.0286	0.1430	[-0.2525,0.3097]	0.8416	
Thought Suppression → WI	-0.0377	0.1412	[-0.3153,0.2398]	0.7894	
**Indirect effects**					
Emotional impact	-0.0543	0.0632	[-0.1824,0.0654]		
Interference	0.0422	0.0589	[-0.0629,0.1693]		
Egodystonicity	0.0043	0.0235	[-0.0422,0.0556]		
Thought Suppression	-0.0064	0.0265	[-0.0626,0.0452]		

Note: Data are unstandardized coefficients, standard errors (SE), confidence intervals (CIs), *p* and R^2 values of the parallel multiple mediation analysis.
